# Hybrid Biomechanical Design of Dental Implants: Integrating Solid and Gyroid Triply Periodic Minimal Surface Lattice Architectures for Optimized Stress Distribution

**DOI:** 10.3390/jfb16020054

**Published:** 2025-02-09

**Authors:** Dawit Bogale Alemayehu, Masahiro Todoh, Song-Jeng Huang

**Affiliations:** 1 Division of Human Mechanical Systems and Design, Graduate School of Engineering, Hokkaido University, Sapporo 060-8628, Japan; zetseatdawit2018@gmail.com; 2 Division of Mechanical and Aerospace Engineering, Faculty of Engineering, Hokkaido University, Sapporo 060-8628, Japan; todoh@eng.hokudai.ac.jp; 3 Department of Mechanical Engineering, National Taiwan University of Science and Technology, Taipei 10607, Taiwan

**Keywords:** finite element method, implantology, biomechanical, additive manufacturing, dental implant, osseointegration, hybrid

## Abstract

Background: Dental implantology has evolved significantly since the introduction of additive manufacturing, which allows for the reproduction of natural bone’s porous architecture to improve bone tissue compatibility and address stress distribution issues important to long-term implant success. Conventional solid dental implants frequently cause stress shielding, which compromises osseointegration and reduces durability. Aim: The current research proposes to examine the biomechanical efficacy of fully and hybrid gyroid triply periodic minimum surface (TPMS) latticed implants across different cell sizes to optimize stress distribution and improve implant durability. Methods: This study evaluates six fully and hybrid gyroid (TPMS) latticed implants, including fully latticed designs with three cell sizes—FLI_111 (1 mm × 1 mm × 1 mm), FLI_222 (2 mm × 2 mm × 2 mm), and FLI_333 (3 mm × 3 mm × 3 mm)—and hybrid gyroid TPMS latticed implants with solid necks in corresponding sizes—HI_111, HI_222, and HI_333. To enhance initial stability, a square-threaded design was added into the bottom part of both fully and hybrid lattice implants. The designs also incorporate anti-rotational connections to enhance fixation, and they undergo a clinical viability comparison with contemporary implants. To improve lattice designs, finite element analysis (FEA) was utilized through nTopology (nTOP 4.17.3) to balance stiffness and flexibility. To examine mechanical performance under realistic conditions, a dynamic mastication loading simulation was conducted for 1.5 s across three cycles. Results: The findings reveal that hybrid implants, particularly HI_222, exhibited improved mechanical characteristics by reducing micromotions at the bone–implant interface, improving osteointegration, and attaining better stress distribution. Conclusions: By addressing stress shielding and boosting implant performance, this work paves the way for personalized implant designs, developing dental technology, and improving clinical results.

## 1. Introduction

The lattice structure, a subset of the TPMS (Triply Periodic Minimal Surface) family, has a highly interwoven and sophisticated architecture, with the gyroid TPMS lattice standing out as a particularly notable subtype [1,2,3]. In recent years, a unique type of sophisticated biomimetic lattice structure (known as TPMS) has received a lot of interest in the field of bone tissue engineering. These structures are distinguished by their distinct periodic geometric variations in three coordinate directions and their zero-mean curvature, making them ideal for the tissue engineering sector [4]. Many industries, particularly those related to dental applications and the broader field of tissue engineering, praise this structure for its immense potential [5]. Its intricate and precision-driven design is ideal for incorporation into dental implants, where rigorous standards are required [6,7]. The gyroid lattice enables the precise adjustment of sophisticated geometric arrangements to meet specific medical requirements. This potential is the result of taking advantage of cutting-edge 3D printing technology [8,9].

The porous structure of the gyroid lattice makes it ideal for tissue engineering applications [10,11,12]. These structures’ particular porosity promotes vascularization and cell proliferation, both of which are required for implants to successfully integrate with surrounding biological tissues [13,14,15]. Porous implant shapes, such as gyroid and Voronoi designs, promote osteointegration by optimizing stress distribution and opening up channels for improved blood flow and nutrient transfer, as shown in bone scaffolds and hip implants. These characteristics make porous designs biomechanically and physiologically superior to solid implants in clinical applications [16,17]. Throughout the 3D printing process, the precise regulation of this porosity enables excellent bone ingrowth and osseointegration, crucial for the long-term success of dental implants [18,19,20]. Furthermore, the intrinsic complexity of lattice geometry, rather than being a disadvantage, is a tremendous benefit in the age of additive manufacturing [11,21,22].

The higher porosity of the gyroid TPMS lattice improves both structural integrity and osseointegration where the implant meets the bone [23,24,25]. This feature is crucial for the long-term success of dental implants by promoting better bone growth and stability [26,27]. These structures’ versatility in responding to many forms of advanced manufacturing, particularly additive manufacturing, emphasizes their usefulness even more. Since the introduction of additive manufacturing technology, the capacity to produce intricate and complicated structures such as gyroid TPMS lattices has transformed dental implant design [11,28]. Among the many current manufacturing processes, laser powder bed fusion (L-PBF) and other metal 3D printing techniques stand out. L-PBF, in particular, offers a highly effective solution for creating implants with detailed internal architectures [29]. Its capacity to deposit material precisely layer by layer makes it appropriate for both tissue engineering scaffolds and complicated dental implants [30,31,32]. Smaller lattice cell sizes increase stress distribution and mechanical dependability, addressing limits in manufacturability and load-bearing capability encountered with larger cell sizes, making them ideal for hybrid dental implant designs [4,33]. Commercially pure titanium and its alloys are the preferred materials for these applications because they are known for their superior mechanical qualities and biocompatibility, making them perfect for medical use [34,35,36].

Usually, exact equations regulate the design and characteristics of lattice structures, which are complex mathematical creations [37,38,39]. These elaborate designs are not just theoretical, but also have practical applications in a variety of sectors, owing to the employment of advanced computational tools. A recent study used MATLAB (MATLAB version: 9.13.0 (R2022b), Natick, Massachusetts), a robust numerical computing environment, to develop code that explicitly specifies and manipulates the geometry of lattice structures [40], enabling a high degree of customization and accuracy. Furthermore, intuitive design and engineering software tools, such as ANSYS SpaceClaim (ANSYS SpaceClaim 2024R1, Cybernet, Chiyoda-ku, Tokyo, Japan) provide user-friendly interfaces for building and updating 3D models, allowing designers to explore complicated lattice geometries more easily [41]. Computer-aided design (CAD) software, such as PTC Creo Parametric version 8, provides robust tools for detailed design and engineering, which is critical for integrating specific mechanical requirements of lattice structures into functional products [42,43], whereas the nTopology (nTOP 3.12.1) optimization software stands out for its ability to fine-tune designs for performance criteria, making it invaluable in optimizing architectural efficiency [44]. The gyroid lattice implant demonstrates how these various software applications may work together to create a very practical and unique design [11,12,45]. We used these sophisticated techniques to create the basic CAD model for this implant, showcasing the flexibility and adaptability of lattice design across diverse applications [46,47,48,49]. Engineers and designers can use sophisticated software solutions to push the boundaries of what is possible in lattice fabrication, resulting in breakthroughs in fields ranging from aerospace to biomedical engineering, emphasizing the collaborative role of various advanced software in refining and realizing these designs [48,50,51,52].

In research, the Finite Element Method (FEM) is a well-known numerical technique that not only reduces material waste but also saves money and time, improving overall efficiency in engineering projects [53,54,55,56,57,58]. Most FEM research has primarily focused on using quasi-static loading analysis to effectively simulate the compression behavior of lattice structures, which is critical for evaluating their structural integrity under varying load situations [56]. However, despite its widespread use, only a few studies have investigated the possibilities of dynamic explicit analysis [59,60]. This approach has demonstrated high dependability in confirming experimental results by precisely mimicking real-world dynamic impacts and stress conditions that lattice structures may experience [61,62,63,64]. The effectiveness of dynamic explicit analysis in generating informative data and exposing unique response characteristics under dynamic loading situations offers a potential field of study that is mostly unexplored [57]. Given this context, the current work aims to delve deeper into this understudied topic.

The main goal of this study is to use explicit dynamic finite element analysis to fully understand how initially fully latticed dental implants behave mechanically when they are under dynamic load. Based on these analytical findings, the project intends to systematically create an optimum dental implant with a novel hybrid design. This design combines a solid neck with a lattice construction for the rest of the implant, with a focus on improving biomechanical compatibility and structural stability. The hybrid structure aims to minimize stress accumulation in crucial areas of the implant, particularly the neck and first thread, which have the highest risk of failure. Using the FEM, this study thoroughly assesses stresses and strains inside the implant structure, providing a solid basis for improving the suggested design. This extensive evaluation not only improves the ability to comprehend stress distribution patterns, but it also allows for implant optimization to better resist physiological loading conditions. We believe that the inclusion of a square-threaded region would improve the hybrid lattice structure’s ability to optimize mechanical characteristics and lower stress intensities more effectively than the fully latticed design, especially in regulating micromotions under cyclic masticatory loading. Finally, the goal of this research is to apply these enhanced analytical insights to actual implant design advances, therefore creating new standards in dental implantology and providing considerable advantages to patient care.

## 2. Materials and Methods

### 2.1. Assembly CAD Model and 2D Drawing

**Figure 1:**
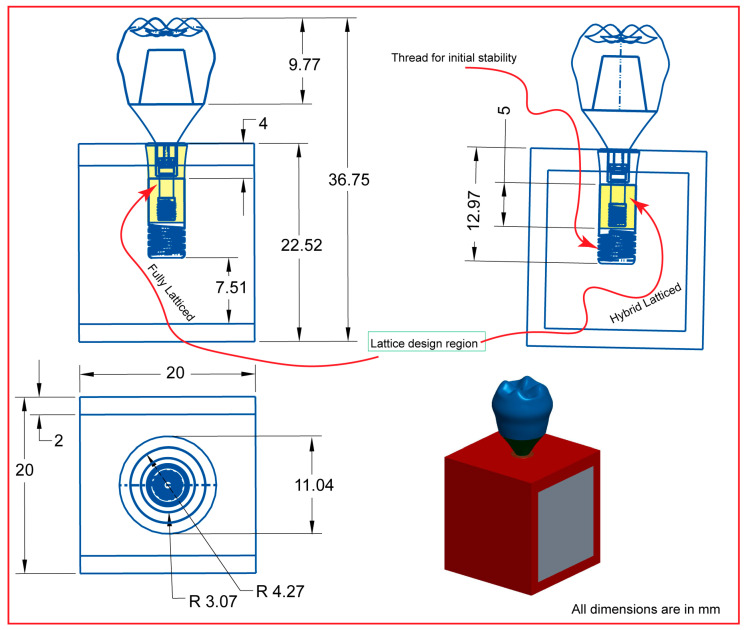
Assembled implant system 3D and 2D drawings.

**Figure 2:**
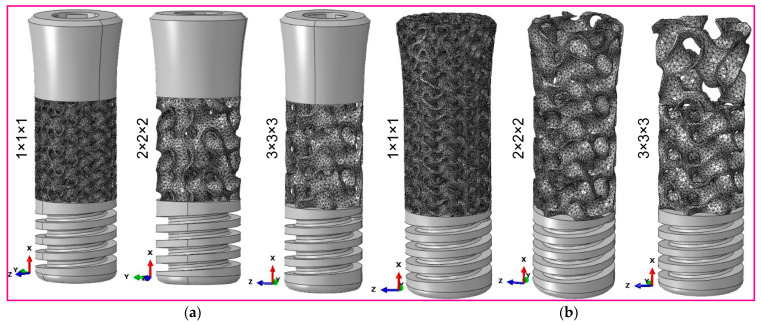
Dental implant models with (**a**) fully and (**b**) hybrid gyroid lattice configurations.

Creo Parametric version 8.0 software precisely designed the dental implant system’s primary components, including cancellous and cortical bone structures. Each model used an implant length of 13 mm, a diameter of 4.1 mm, and a pitch of 0.8 mm, based on the clinically validated Straumann^®^ Standard Plus (SP) dental implant (Straumann, Basel, Switzerland) with biomechanical relevance [57]. As seen in Figure 1, the software assisted in the creation of both two-dimensional drawings and three-dimensional models, ensuring that all dimensions were appropriately displayed. The primary focus of this design effort is the implant itself, which will incorporate a gyroid lattice structure in various configurations, including both fully and hybrid latticed dental implant designs. This method not only enhances the implant’s visibility in its functional environment, but also enables precise design modifications specifically tailored to integrate the gyroid lattice structure efficiently. Both fully and hybrid lattice implants now have a square-threaded section on the bottom to improve initial stability and clinical survivability (refer to Figure 1 and Figure 2). This characteristic enhances primary stability during implant placement, especially in softer bone types. Furthermore, we added an anti-rotational connection to the prosthetic screw-retaining mechanism to limit rotational movement and maintain a stable, long-term fit between the implant and prosthetic components.

### 2.2. Dental Implant Lattice Design

Figure 2 shows a dental implant filled with a gyroid lattice structure in two different layouts. These layouts include fully and hybrid latticed dental implant designs that use three different cell sizes, which are 111, 222, and 333. The three-dimensional (3D) model employed in this work was based on a rectangular slice of cortical and trabecular bone extracted from a human jaw. We methodically created the original implant design using Creo Parametric version 8.0 software, a powerful CAD application. We accurately established the specific dimensions of each model, which included a length of 14.32 mm, a diameter of 4.1 mm, and a wall thickness of 0.8 mm. This accuracy in design parameters guarantees that the implants are ideally structured to fulfill particular biomechanical criteria, and allows for the examination of structural variations dependent on cell size within lattice designs.

### 2.3. Dental Implant FE-Meshing and Mechanical Properties

**Figure 3:**
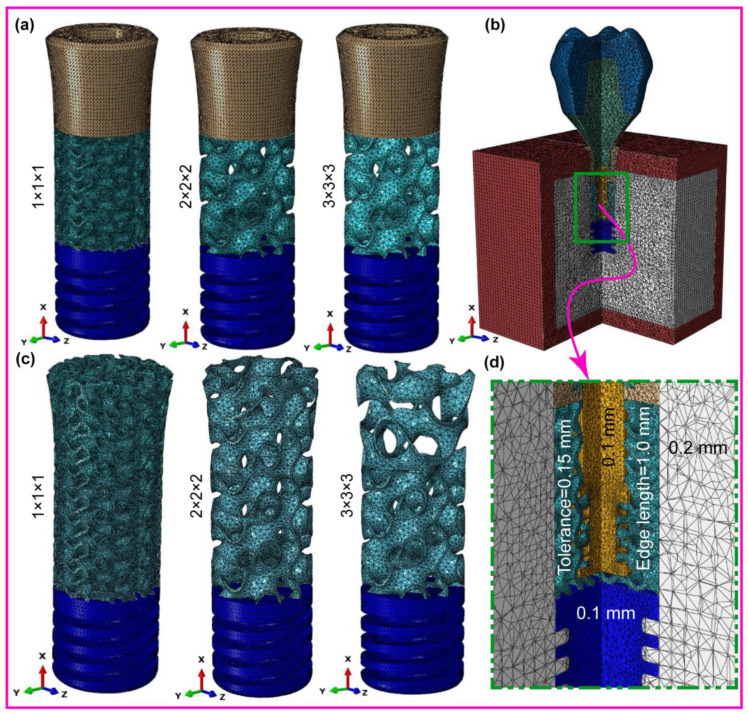
FE mesh for (**a**) fully latticed implant, (**b**) assembled dental implant, (**c**) hybrid latticed implant, and (**d**) zoomed-in FE mesh details indicated by rectangle.

Figure 3 illustrates the process of saving the dental implant configurations within the system components as SAT file extensions. We then export these files to nTopology (nTOP 4.17.3) for the creation of finite element meshes. Both the fully latticed and hybrid latticed dental implants use three distinct cell sizes—111, 222, and 333. We mesh each model using 0.1 mm finite element (FE) sizes and 1.0 mm edge length. Here, 0.1 mm elements mesh the retaining screw and solid neck in the hybrid lattice. The compact bone and spongy porous bone, which are crucial for implant stability, are more finely meshed around the implant hole, improving the reliability of stress evaluation in these key locations. On the other hand, we mesh the remaining areas with a coarser element size of 0.3 mm to effectively manage computational resources while maintaining model fidelity. This method of precise meshing ensures the appropriate design of every dental implant system component for structural study, enabling a deeper exploration of the biomechanical characteristics present in both fully and hybrid latticed systems. The physical properties of the materials for the dental implant, its components, and bones are listed in Table 1 [49,57].The mesh statistics for the dental implant, its components, and assembly models are presented in Table 2. This method intends to create a biomimetic scaffold that not only closely resembles the architecture of natural bone, but also maximizes the implant’s biomechanical performance by incorporating strategic design changes. 

### 2.4. Dynamic Mastication Loading and Boundary Conditions

**Figure 4:**
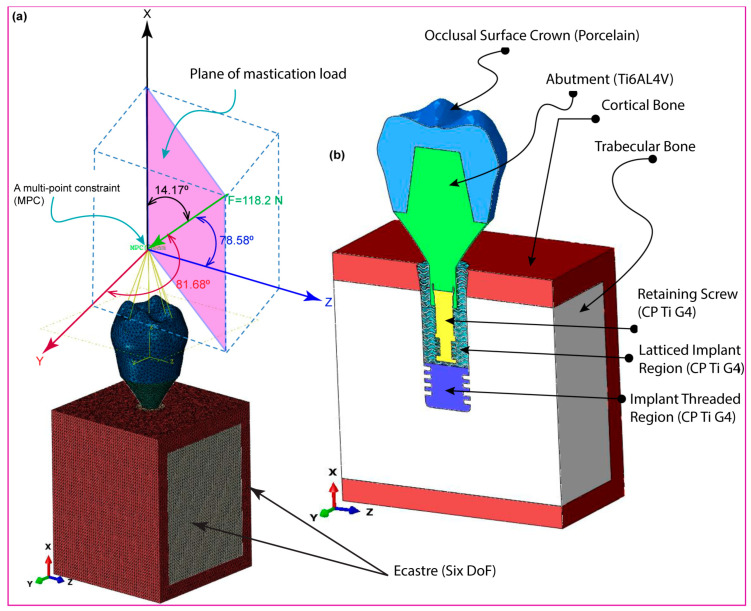
Dental implant system, (**a**) dynamic mastication loading for 0.5 s with 2 Hz in a single cycle, and (**b**) its components.

Figure 4a,b show the constructed implant system under dynamic load, using one of the two latticed implant designs investigated in this work. Dynamic explicit analysis was used to mimic real-world events that might occur during the implant’s clinical usage. This picture (Figure 4a) shows the implant when it is loaded at an angle of 78.58 degrees, with forces acting in the mesiodistal, axial, and buccolingual directions. We subjected the occlusal crown surface to stresses from three different directions—mesiodistal, buccal-lingual, and apical—to evaluate the biomechanical response of the dental implant under multi-axial dynamic oblique loading. We strategically delivered force magnitudes of 23.4 N, 17.1 N, and 114.6 N, converging at an ill-defined reference point 3 mm from the occlusal surface using a multi-point constraint (MPC) technique. We created this arrangement to simulate the complex pressures experienced during clinical mastication, resulting in an equivalent force of 118.2 N, inclined at 75.8 degrees relative to the occlusal plane [49,57]. We reduced the explicit dynamic loading to a slower rate to replicate the cyclic stress of mastication, typically detected at 2 Hz over a 0.5 s interval. Furthermore, carefully created boundary conditions mimicked real-world restrictions at the implant–mandible interface. A six-degree-of-freedom (DoF) Encastre boundary condition held the implant securely in all three spatial dimensions (X, Y, and Z). This made it possible to replicate the stress distribution exactly in many directions. With this thorough setup, exact measurements of compressive and tensile stresses inside the implant system could be made using finite element analysis with ABAQUS/CAE 2021. This made the study more reliable.

### 2.5. Contact Definition

This approach adequately predicts stress and strain distributions at the bone–implant interface, which is critical for assessing implant mechanical performance under a variety of loading circumstances. Nonlinear contact zones were established at one of the crucial interfaces—for the implant–bone interactions with bone–bone contact, we used a Tie contact constraint, as there should be no relative motion between the cortical and trabecular bone surfaces. The contact analysis demonstrated how load and deformation are transmitted between various components. The cortical bone–implant contact was given a friction coefficient (µ) of 0.65 [65], while the cancellous bone–implant interface was assigned a value of 0.77 [66]. Furthermore, the connections between the crown and abutment, the abutment and screw-retaining mechanism, and the implant and screw-retaining system were designated as bonded connections to provide secure and stable interactions between these components for maximum load transfer and structural integrity.

**Table 1:** Mechanical properties of materials for finite element analysis [49,57,67].

Materials	Young’s Modulus E (MPa)		Poisson’s Ratio ν		Density (g/cm^3^)	Strength (MPa)
Cortical bone *	E_x_	12,600	ν_xy_	0.3	1.79	190
	E_y_	12,600	ν_yz_	0.253		
	E_z_	19,400	ν_xz_	0.253		
			ν_yx_	0.3		
			ν_zy_	0.39		
			ν_zx_	0.39		
Cancellous bone *	E_x_	1148	ν_xy_	0.055	0.45	10
	E_y_	210	ν_yz_	0.01		
	E_z_	1148	ν_xz_	0.322		
			ν_yx_	0.01		
			ν_zy_	0.055		
			ν_zx_	0.322		
Ti-6Al-4V *	115,000		0.33		4.42	830
Porcelain	68,900		0.28		2.44	145
Titanium grade 4 *	110,000		0.34		4.5	550

* The vectors of x, y and z are the infero-superior (axial), mesiodistal, and buccolingual directions, respectively. Implant threaded, implant latticed regions and screw retaining = Titanium grade 4. Abutment = Ti-6Al-4V. Crown = Porcelain.

### 2.6. Design and Manufacturing Process

**Figure 5:**
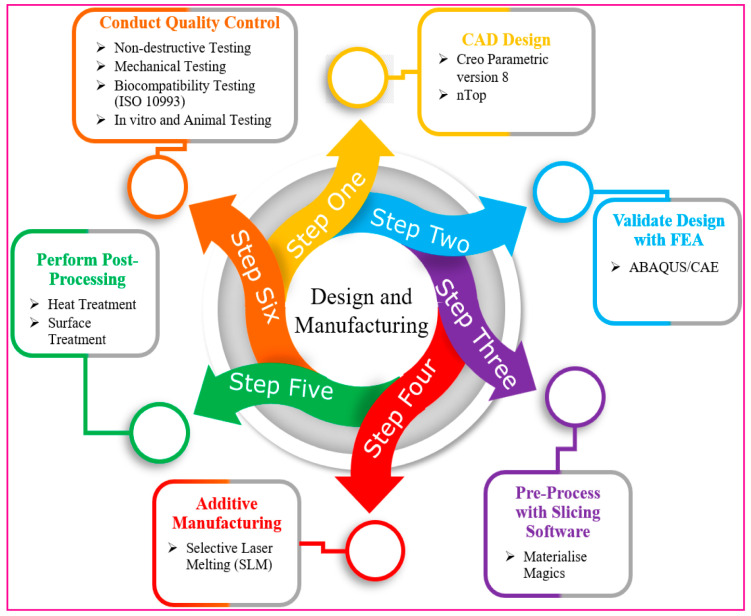
Workflow for the design and manufacturing steps.

We used several integrated stages to develop and manufacture the suggested dental implants (refer to Figure 5). As illustrated in Figure 5, this study focuses on steps 1 to 3, while steps 4 to 6 serve as a roadmap for future research, outlining the next stages beyond finite element analysis. Two novel designs were created—a totally TPMS gyroid lattice structure with a square-threaded bottom area and a hybrid design with a solid neck, TPMS lattice middle portion, and square-threaded lower region. The CAD models were made in Creo Parametric version 8.0, and the lattice structures were made in nTop 4.17.3 so that the size of the pores and the thicknesses of the struts could be controlled by our parameters. The validation was carried out using the ABAQUS/CAE 2021 finite element analysis (FEA) program, which replicated physiological loading conditions to assess stress distribution, durability, and structural integrity. The Materialise Magics slicing technique improved layer thickness (30–50 μm) and incorporated support strategies for difficult shapes. Selective Laser Melting (SLM) with Titanium Grade 23 (Ti-6Al-4V ELI) was used in additive production, with validated parameters ensuring structural accuracy. Heat treatment at 650 °C for stress relief was followed by surface treatments such as sandblasting, acid etching, and micro-arc oxidation (MAO) to improve osseointegration and CNC machining for dimensional precision. Non-destructive testing (e.g., X-ray CT scanning) and mechanical testing (compression, fatigue) were used for quality control, in accordance with ISO 14801 [68] standards, as well as biocompatibility assessments according to ISO 10993 [69] criteria. The final sterilization process included gamma irradiation or ethylene oxide gas, with sterile packing guaranteeing compliance with medical equipment laws. This approach, underpinned by thorough testing and validation, illustrates the viability and preparedness of the implants for clinical use. Table 3 provides a comparison of current latticed and previous implant designs, highlighting key differences in implant length, thread type, and lattice structure type. Notably, the table contrasts the use of Gyroid TPMS lattice structures with more traditional designs, such as the BCC and diamond lattices, across various implant configurations [68,69,70,71,72,73].

Design and manufacturing workflow.

**Table 2:** FE mesh statistics of fully and hybrid latticed dental implants, implant components, and their assembly models.

	Crown		Abutment		Screw		Implant		Cortical Bone		Trabecular Bone		Total	
Implant Models	No. Element	No. Node	No. Element	No. Node	No. Element	No. Node	No. Element	No. Node	No. Element	No. Node	No. Element	No. Node	No. Element	No. Node
FLI_111	33,454	6725	20,571	4405	85,696	18,875	731,804	192,853	315,057	63,003	392,084	72,096	1,578,666	357,957
FLI_222	33,454	6725	20,571	4405	85,696	18,875	520,103	116,644	315,057	63,003	392,084	72,096	1,366,965	281,748
FLI_333	33,454	6725	20,571	4405	85,696	18,875	155,642	37,459	315,057	63,003	392,084	72,096	1,002,504	202,563
HI_111	33,454	6725	20,571	4405	85,696	18,875	706,444	170,246	315,057	63,003	392,084	72,096	1,553,306	335,350
HI_222	33,454	6725	20,571	4405	85,696	18,875	598,523	127,878	315,057	63,003	392,084	72,096	1,445,385	229,982
HI_333	33,454	6725	20,571	4405	85,696	18,875	244,346	52,473	315,057	63,003	392,084	72,096	1,091,208	217,577

FLI_111, FLI_222, and FLI_333 represent fully latticed implants with cell sizes of 1 mm^3^, 8 mm^3^, and 9 mm^3^, respectively, while HI_111, HI_222, and HI_333 denote hybrid latticed implants with cell sizes of 1 mm^3^, 8 mm^3^, and 9 mm^3^, respectively.

**Table 3:** Comparison table for current latticed and previous implant design.

Implant Length	Thread Type	Lattice Structure Type	Reference
Long	Square	Gyroid (TPMS)	Current paper
Ultra-Short	Buttress	Diamond lattice	[70]
Long	V-screw	Gyroid and body center cubic octahedron (BCCO)	[71]
Long	Fully latticed	Body center cubic octahedron (BCC)	[72]
Long	Fully latticed	Diagonal lattice	[73]
Long	V-thread	Body-centered cubic (BCC)	[74]
Long	V-thread	Diamond lattice	[75]
Long	V-thread	Gyroid-TPMS lattice	[74]

## 3. Results

### 3.1. Cyclic Mastication Loading

**Figure 6:**
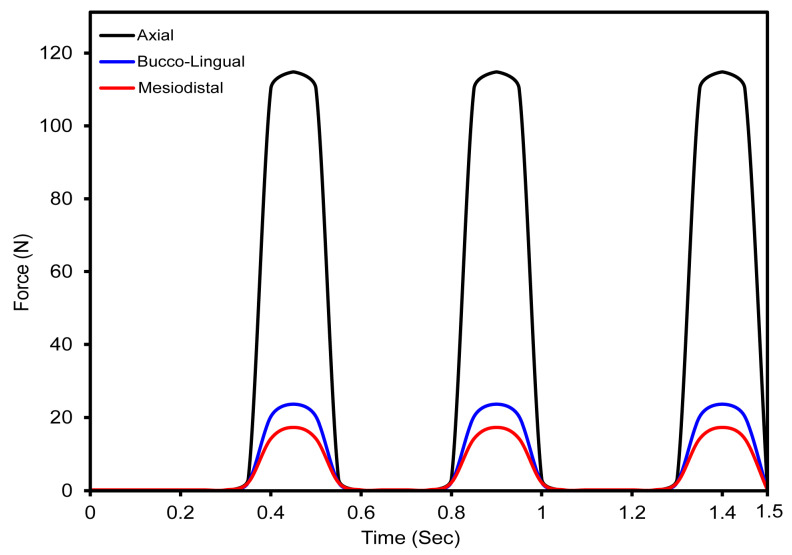
Oblique dynamic loading distribution at 118.2 N in buccal–lingual, axial, and mesiodistal directions over a 0.5 s mastication cycle.

Figure 6 shows how an oblique dynamic load of 118.2 N is applied to the occlusal surface of a dental crown in the buccal–lingual, axial, and mesiodistal directions. Human mastication frequencies generally vary from 0.94 Hz to 2.17 Hz [76], which corresponds to the physiological chewing patterns investigated in this work. This loading replicates a 2 Hz mastication cycle, which accurately reproduces the dynamic stress patterns over a 1.5 s period. The figure depicts increasing load variation throughout the mesiodistal, buccal–lingual, and apical orientations, revealing a smooth shift in load amplitude. The use of multi-point constraints (MPC) with a reference point as the master control considerably improved the accuracy of masticatory force replication, reflecting the complex dynamics found in clinical situations. This technique verified that the load distribution closely matched the parameters established in earlier research [32], hence proving the validity of the simulated masticatory forces on both completely and hybrid latticed dental implants. The cell sizes of the fully latticed designs were FLI_111, FLI_222, and FLI_333, while the hybrid latticed designs were HI_111, HI_222, and HI_333. The results of this explicit dynamic loading and the related boundary conditions closely match those of previous findings, demonstrating the simulation’s resilience in representing real-world biomechanical phenomena.

### 3.2. Mesh Sensitivity Analysis

**Figure 7:**
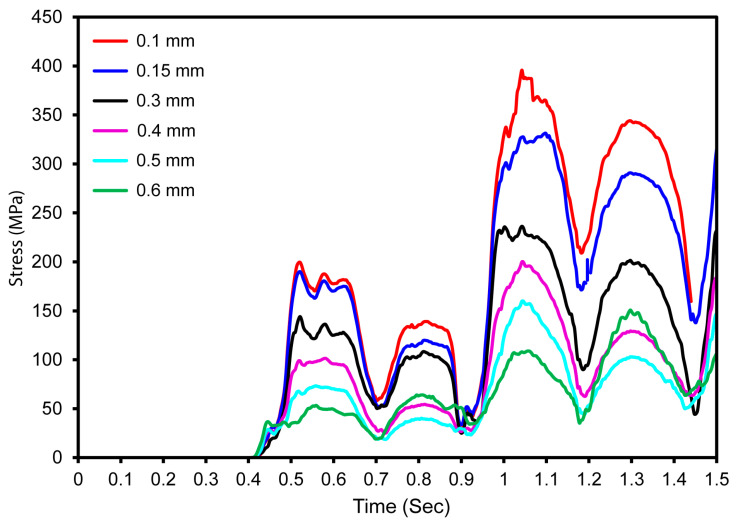
Fully latticed implant with a cell size of 333 mesh sensitivity analysis, showing maximum stress against time with different element sizes.

We performed a mesh sensitivity study on a fully latticed dental implant with 333 cells. This cell size was chosen because it is computationally efficient and requires less simulation time (refer to Figure 7). The walls of the TPMS lattice with a cell size of 333 require less travel distance to reach the next layer than lattices with cell sizes of 111 and 222, which require more travel distance. We investigated element sizes ranging from 0.1 mm to 0.5 mm to see how maximum stress varies with mesh size. The stress vs. time graphs shows minimal differences in maximum stress between 0.1 mm and 0.15 mm element sizes (refer to Figure 6). The ideal mesh size for a fully latticed dental implant with a cell size of 333 is 0.1 mm, based on the precision required for finite element analysis and the measured peak stress of 396 MPa.

### 3.3. Maximum von Mises Stress in Dental Implant Assembly

#### 3.3.1. Fully Latticed Dental Implant

**Figure 8:**
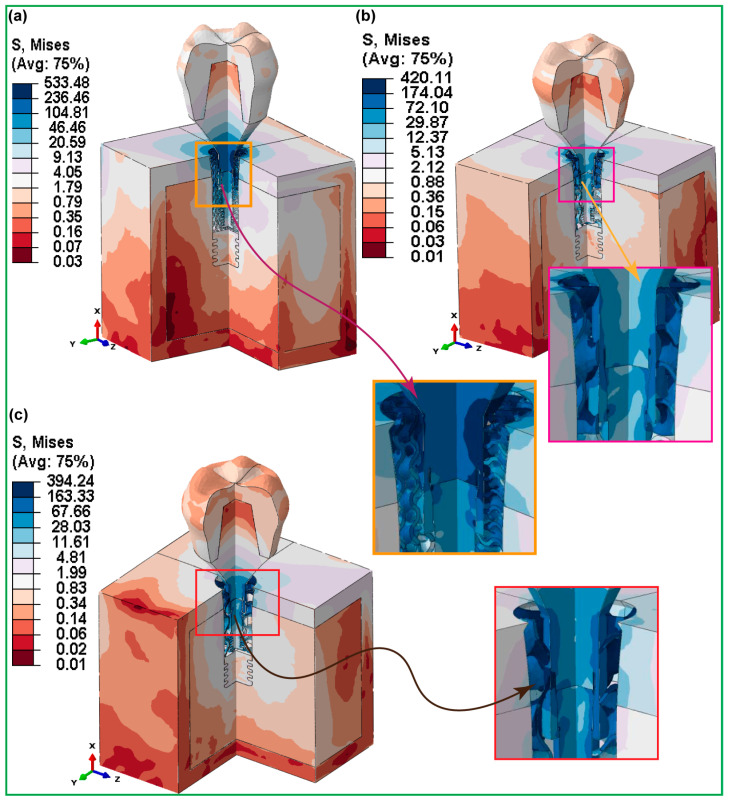
FEA Stress Contour Plots for von Mises stress distributions in assembled and cross-sectioned views of three fully gyroid latticed dental implants with varying cell sizes: (**a**) FLI_111, (**b**) FLI_222, and (**c**) FLI_333.

Figure 8 presents the highest von Mises stresses that were found in three totally gyroid latticed dental implants with different-sized cells. The implant with the smallest cell size, FLI_111 (1 × 1 × 1), showed the greatest stress, measured at 533.48 MPa. This degree of stress indicates a more concentrated load-bearing capability, perhaps because of the narrower lattice structure, which may give less distribution room for stress. In comparison, the FLI_222 implant, with a cell size of 2 × 2 × 2, showed a decrease in von Mises stress of 420.11 MPa. This suggests increased stress distribution capabilities, most likely due to the larger cell size, which allows for more efficient mechanical force distribution over the implant structure. The biggest cell size studied, FLI_333 (3 × 3 × 3), had the lowest von Mises stress of 394.24 MPa. This further decline implies that the implant’s capacity to disperse mechanical stress increases with cell growth, leading to reduced total stress levels. Stress concentrations were highest around the implant–bone contact areas, especially near the holes in the cortical and cancellous bone, as well as the implant neck. Furthermore, the highest stress was focused at important interfaces such as the crown–abutment, abutment–implant, screw–retaining, and abutment–implant contact areas.

#### 3.3.2. Hybrid Latticed Dental Implant

**Figure 9:**
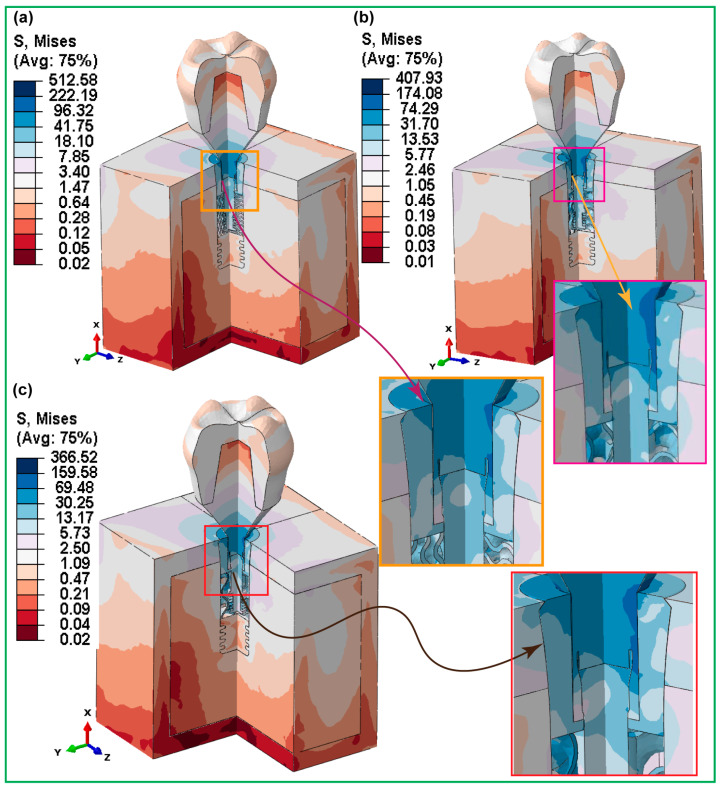
FEA stress contour plots showing maximum von Mises stress distributions in the assembled and detailed rectangular sections of the hybrid latticed dental implant system with three varied cell sizes: (**a**) HI_111, (**b**) HI_222, and (**c**) HI_333.

Figure 9 displays how the stresses behave under simulated biting forces for the highest von Mises stresses in three hybrid gyroid latticed dental implants, whose cell sizes varied. The HI_111 implant, with a 1 × 1 × 1 cell size design, showed a maximum stress of 512.58 MPa. However, HI_222 showed a reduced stress level (407.93 MPa) due to its larger cell size of 2 × 2 × 2. Likewise, the largest implant in terms of cell size, HI_333 (3 × 3 × 3), had a lower stress value of 366.52 MPa. These findings indicate that among hybrid latticed designs, the implant with a smaller cell size suffered the most stress, while both smaller and larger cell topologies resulted in lower stress levels.

In contrast, in the case of fully latticed implants, as cell size increased from FLI_111 to FLI_333, the von Mises stress decreased consistently, demonstrating that larger cell sizes result in better stress distribution. The hybrid latticed implants, on the other hand, showed a non-linear pattern, with the largest stress recorded in the implants with medium cell size (HI_222). This was different from the trend seen in the fully latticed implants. This variation emphasizes the complicated relationship between lattice architecture and stress distribution in dental implants. Stress concentrations were highest around the implant–bone contact areas, especially near the holes in the cortical and cancellous bone, as well as the implant neck. Furthermore, the highest stress was focused at important interfaces such as the crown–abutment, abutment–implant, screw–retaining, and abutment–implant contact areas.

#### 3.3.3. Fully and Hybrid Latticed Dental Implant and Retaining Screw

**Figure 10:**
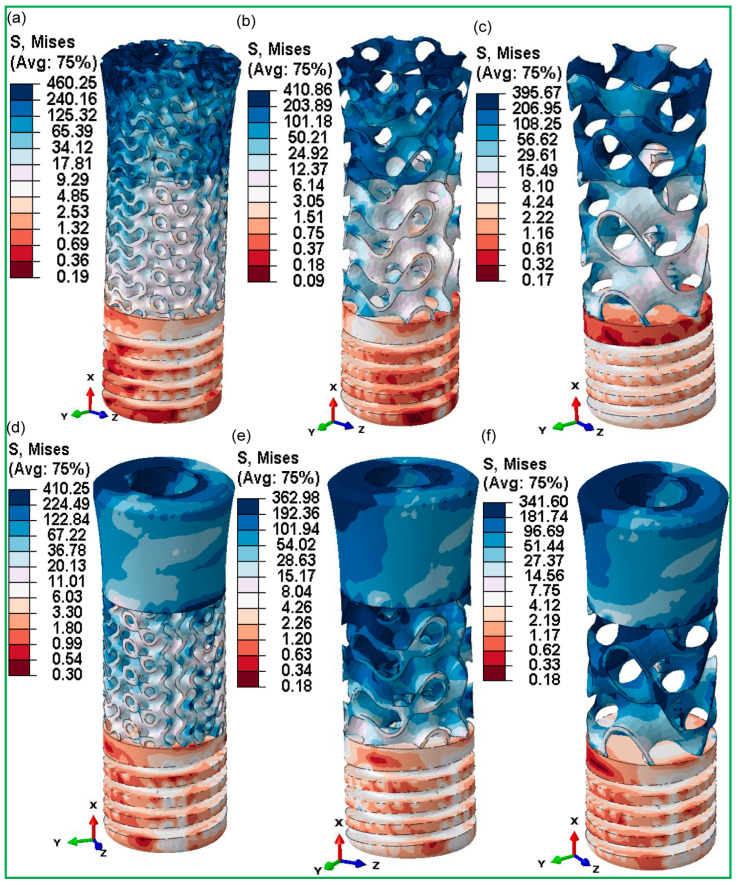
Maximum von Mises stress distribution for fully latticed implants—(**a**) FLI_111, (**b**) FLI_222, (**c**) FLI_333; and for hybrid latticed implants—(**d**) HI_111, (**e**) HI_222, (**f**) HI_333.

Figure 10 shows a comparison of the maximum von Mises stresses for completely gyroid latticed and hybrid gyroid latticed dental implants, as well as their corresponding retention screws, across various cell sizes. Fully latticed implants show a clear pattern in which von Mises stress reduces with increasing cell size. The FLI_111 implant, with the lowest cell size of 1 × 1 × 1, showed the maximum stress at 460.25 MPa. The high stress level indicates that smaller lattice cells may concentrate stress more than larger ones. The FLI_222 and FLI_333 implants, with cell sizes of 2 × 2 × 2 and 3 × 3 × 3, respectively, demonstrated reduced stress values of 410.86 MPa and 395.67 MPa. As the cell size rises, the stress distribution over the implant structure improves, possibly increasing the implant’s endurance and performance.

**Figure 11:**
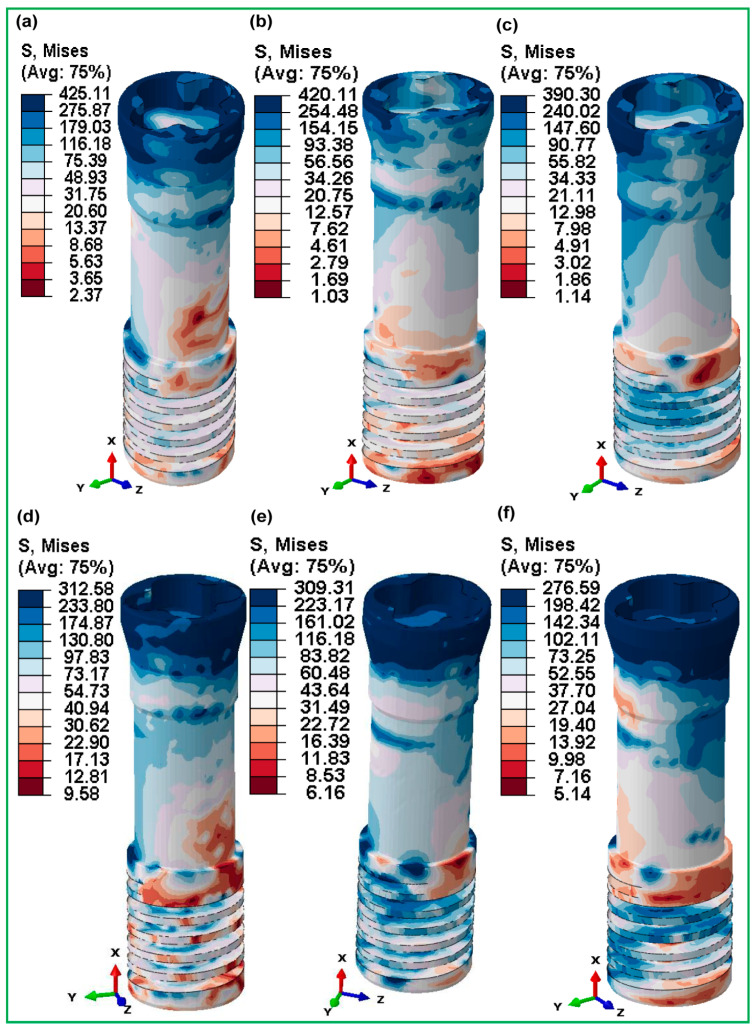
Maximum von Mises stress distribution in retaining screw for various cell sizes—(**a**) FLI_111, (**b**) FLI_222, (**c**) FLI_333 for fully latticed implants, and (**d**) HI_111, (**e**) HI_222, (**f**) HI_333 for hybrid latticed implants.

The retention screws associated with these implants showed similar stress patterns (refer to Figure 11), with the FLI_111 screw having the maximum stress at 425.11 MPa, which subsequently decreased in the FLI_222 and FLI_333 screws to 420.11 MPa and 390.30 MPa, respectively. This pattern highlights the impact of cell size on the mechanical stability of not just implants, but also essential components like screws, which play an important role in the whole implant system. In contrast, the hybrid latticed implants showed a more variable pattern of stress distribution. HI_111, with a cell size of 1 × 1 × 1, had a stress of 410.25 MPa, which is lower than its fully latticed counterpart but still considerable. The HI_222 and HI_333 implants reported values of 362.98 MPa and 341.60 MPa, respectively, demonstrating a less consistent drop in stress with increasing cell size than the fully latticed models. The stresses in the retaining screws for these hybrid models also varied, with the HI_111 screw exhibiting significantly lower stress at 312.58 MPa compared to its fully latticed counterpart, implying that the hybrid design may affect stress distribution differently, possibly due to variations in structural integrity or load-bearing pathways within the implant.

#### 3.3.4. Fully and Hybrid Latticed in Cortical and Cancellous Bone

**Figure 12:**
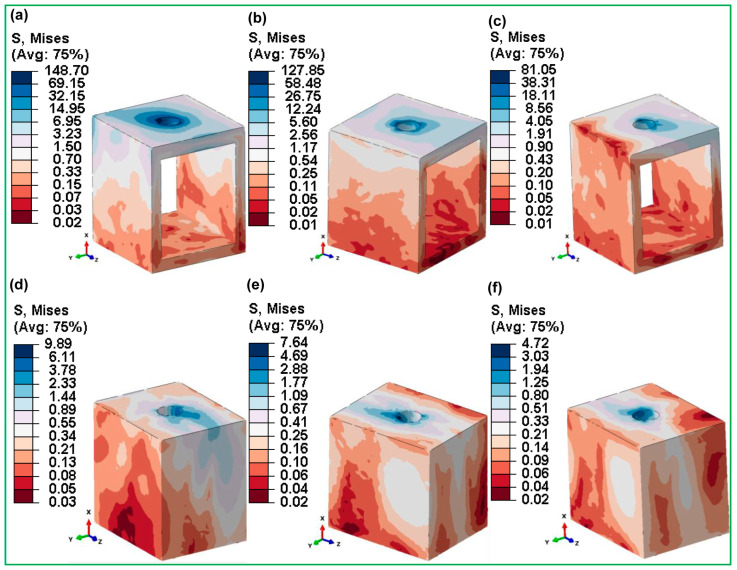
Maximum von Mises stress distributions for fully gyroid latticed dental implants across various cell sizes in cortical bone—(**a**) FLI_111, (**b**) FLI_222, (**c**) FLI_333—and cancellous bone—(**d**) FLI_111, (**e**) FLI_222, (**f**) FLI_333.

Figure 12 shows in detail the highest von Mises stresses in the cortical and cancellous bones that are connected to fully hybrid latticed dental implants with various cell sizes. The von Mises stress results for the fully latticed implants (FLI_111, FLI_222, and FLI_333) show considerable differences depending on cell size and bone type. FLI_111 showed the maximum stress in the cortical bone at 148.70 MPa, indicating a significant concentration of stress, especially in the implant–bone interface around the neck, where the implant and bone are in close contact. This region is crucial because it absorbs the majority of mechanical stresses during mastication. In contrast, FLI_333 exhibits the lowest stress in cortical bone at 81.05 MPa, suggesting a more advantageous stress distribution that may improve implant lifetime and lower the risk of bone resorption.

In Figure 12d, we see that FLI_111 again recorded increased stress at 9.89 MPa for cancellous bone. This is consistent with studies in cortical bone, and highlights a pattern in which smaller cell sizes could aggravate stress concentrations, particularly around the implant neck and hole. Meanwhile, FLI_222 and FLI_333 exhibited lower stresses (7.64 MPa and 4.72 MPa, respectively), suggesting that smaller cell sizes may reduce stress peaks in less dense bone formations.

**Figure 13:**
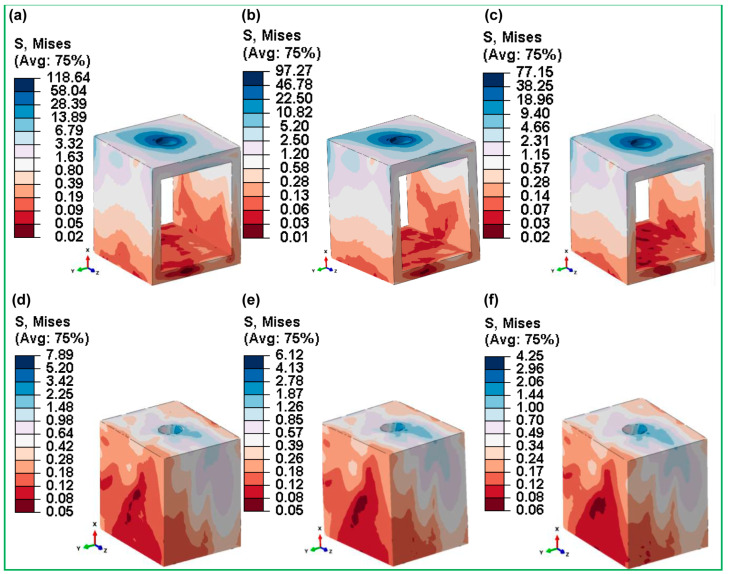
Maximum von Mises stress distributions for hybrid gyroid latticed dental implants across various cell sizes in cortical bone—(**a**) HI_111, (**b**) HI_222, (**c**) HI_333—and cancellous bone—(**d**) HI_111, (**e**) HI_222, (**f**) HI_333.

The stress levels in the cortical and cancellous bones for hybrid gyroid latticed dental implants with a solid neck (HI_111, HI_222, and HI_333) are shown in Figure 13. The HI_111 model, in particular, showed the maximum stress level inside the cortical bone (118.64 MPa). The implant hole, a crucial area wherein the implant closely contacts the bone, concentrates this peak stress. Such a high stress concentration is important because it shows places where the implant’s structure and overall therapeutic performance could be damaged. This suggests that the HI_111 cell size and lattice design may not be as good at distributing stress.

In contrast, the HI_222 implant exhibits substantially lower stress levels, with 97.27 MPa in cortical bone and a remarkably low 6.12 MPa in cancellous bone. These results indicate that HI_222’s lattice architecture and cell size are very efficient in distributing stresses throughout the implant structure. This optimum stress mitigation not only increases the implant’s longevity, but also reduces the likelihood of bone resorption and implant failure, suggesting better biomechanical performance compared to its competition.

#### 3.3.5. Fully and Hybrid Latticed in Crown and Abutment

**Figure 14:**
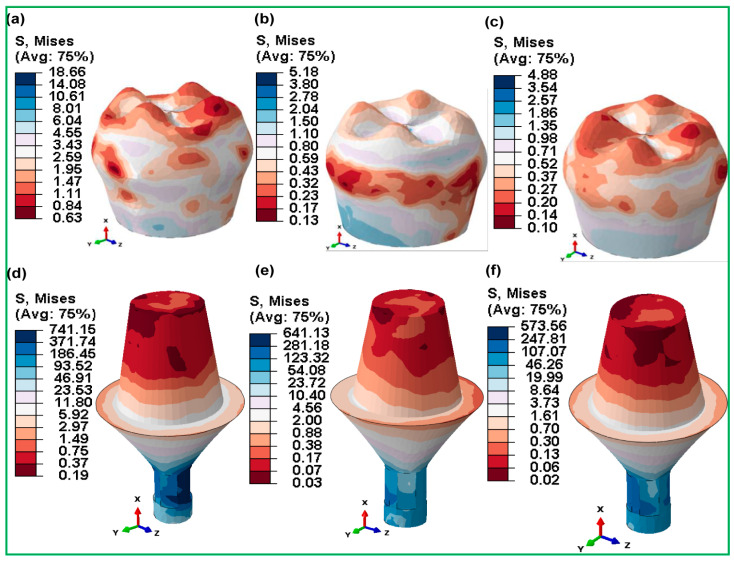
Maximum von Mises stress distributions for fully gyroid latticed dental implants across various cell sizes in crown—(**a**) FLI_111, (**b**) FLI_222, (**c**) FLI_333—and abutment—(**d**) FLI_111, (**e**) FLI_222, (**f**) FLI_333.

**Figure 15:**
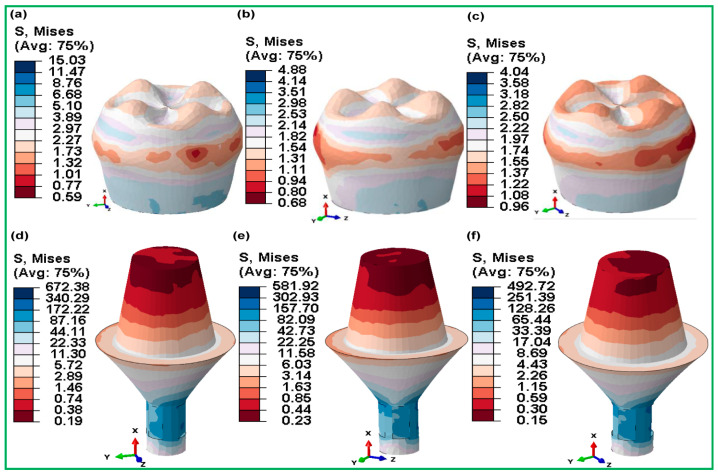
Maximum von Mises stress distributions for hybrid gyroid latticed dental implants across various cell sizes in crown—(**a**) HI_111, (**b**) HI_222, (**c**) HI_333—and abutment—(**d**) HI_111, (**e**) HI_222, (**f**) HI_333.

The highest von Mises stresses can be seen in Figure 14 and Figure 15, which show the crown and abutment parts of fully and hybrid latticed dental implants with three different cell sizes. The stresses of crown components in fully latticed implants (Figure 14) were relatively higher for FLI_111, at 18.66 MPa, and lower for FLI_222 at 5.18 MPa and FLI_333 at 4.88 MPa. On the contrary, the abutment components in the same group showed notably higher stresses—FLI_111 at 741.15 MPa, FLI_222 at 641.13 MPa, and FLI_333 at 573.56 MPa. Conversely, the hybrid latticed implants (Figure 14) demonstrated comparatively lower stress levels in the crown compared to their fully latticed counterparts, with HI_111 at 15.03 MPa, HI_222 at 4.88 MPa, and HI_333 being the lowest at 4.04 MPa.

The abutment stresses in these hybrid models are much higher, resulting in a stiffer dental implant for stress distribution—HI_111 672.38 MPa, HI_222 581.92 MPa, and HI_333 492.72 MPa.

When compared to fully latticed designs, hybrid latticed implants show a more uniform distribution of stresses between the crown and abutment, as well as lower stress levels in the abutment. The hybrid arrangements, in particular, provide much lower stress in the abutment portion as compared to their fully latticed counterparts, suggesting possibly greater mechanical performance under clinical situations. Furthermore, within each configuration, the crown consistently exhibits lower stresses than the abutment, indicating a significant difference in stress management between these two key components of dental implants.

#### 3.3.6. Micromotions in Assembled Dental Implants for Fully and Hybrid Lattices

**Figure 16:**
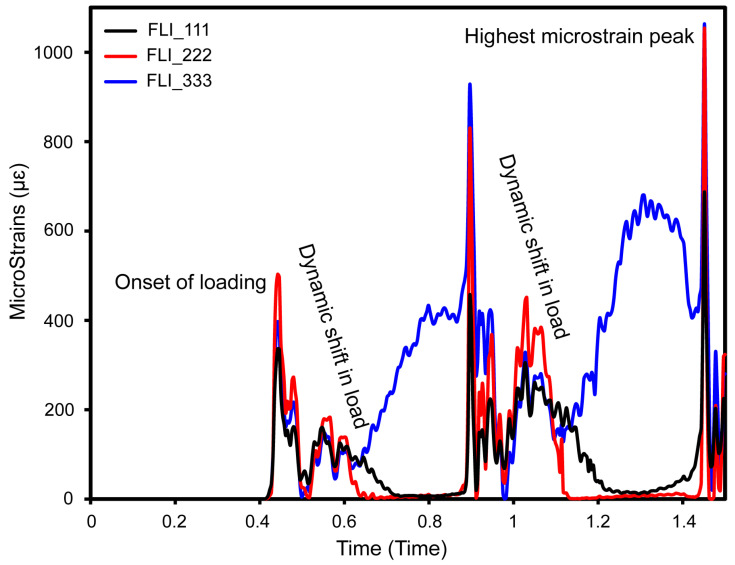
Micro-strain in three cycles of mastication for fully latticed configurations at various cell size (FLI_111, FLI_222, and FLI_333).

Figure 16 shows the micro-strain (µƐ) of fully latticed dental implant setups with different cell sizes (FLI_111, FLI_222, and FLI_333) during 1.5 s over three cycles of mastication with oblique loading. At first, the micro-strain in all three designs was zero, indicating strong initial stability under load. However, after roughly 0.4 s, the micro-strain shifted positively, reaching 450 µƐ for FLI_222 and 415 µƐ for FLI_333. The figure illustrates the behaviors of fully latticed implants (FLI_111, FLI_222, and FLI_333) under oblique dynamic stress during a three-mastication cycle (1.5 s or 2 Hz). The micro-strains steadily increased for all three lattice configurations over the first 0.4 s of loading. Beyond this time, the curves exhibited substantial slope fluctuations, with more stable behavior when nearing 1.5 s. FLI_333 showed the most micro-strain, followed by FLI_222 and the FLI_111, which showed the least movement, illustrating that mechanical responses varied across lattice configurations under the same dynamic situation. These variances indicate that the implants showed varying stiffness and flexibility, which influences their mechanical performance under dynamic mastication stresses.

As the cycle progresses, the micro-strain exhibits a dynamic, cyclic behavior that is consistent with the loading patterns observed during clinical mastication. Specifically, FLI_333 and FLI_222 showed large micro-strains, peaking at 1100 µƐ and 1095 µƐ, respectively. This implies that they are more flexible under stress, which could affect the micromotion at the bone–implant interface. In comparison, FLI_111 showed more controlled micro-strain values of 24 μm and 17 μm, respectively. These changes show the effects of cell size on implant biomechanical performance, with smaller cell sizes (FLI_111) allowing for higher displacement and maybe more micromotion, which may compromise osseointegration and stability. The bigger cell sizes (FLI_333 and FLI_222) exhibited reduced displacement, suggesting a more stable interface under similar loading conditions.

These findings emphasize the importance of lattice structure and cell size in regulating dental implant micro-deformations and subsequent micromotion. Such micromotions are critical for determining the mechanical integration of the implant into the surrounding bone structure, which influences both immediate postoperative stability and long-term osseointegration success.

**Figure 17:**
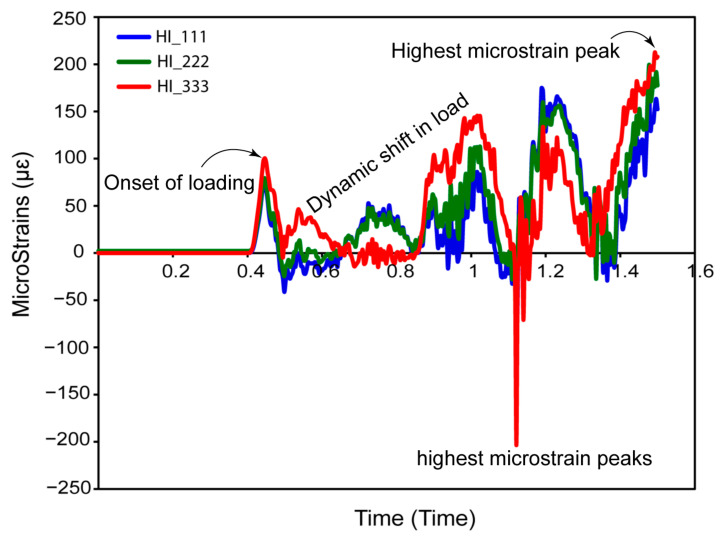
Micro-strain under three cycles of mastication for the hybrid latticed configuration at various cell sizes (HI_111, HI_222, and HI_333).

In Figure 17, we can see in great detail how the micro-deformation responses change in the cortical bone in hybrid latticed dental implants with different cell sizes during the three cycles of 1.5 s of masticatory oblique loading. Initially, all configurations exhibited zero micro-strains at around 0.4 s, indicating early stability. However, immediately after 0.4 s, this peaked at 100 µƐ for HI_333, then further increased to 150 µƐ at 1.0 s, after which the micro-strains shifted towards the negative, significantly rising to 200 µƐ for HI_333 at 1.17 s. Then, the values reached 225 µƐ for HI_333, 190 µƐ for HI_222 and 160 µƐ for HI_111 at around 1.5 s. This implies an initial inward movement under strain, similar to the compressive action seen in masticatory activity.

The micro-strain then entered a dynamic phase with cyclic fluctuations, including peaks and troughs. At around 1.10 s, HI_333 showed the most significant minimal micro-strain at 220 µƐ. The micro-strain follows a cyclic pattern with variable increments of amplitude, demonstrating the implant structure’s durability under cyclic stresses. This cyclic action resembles the normal masticatory process, which is important for determining the implants’ biomechanical compatibility and functional stability.

Comparing these findings to those from Figure 14 for fully latticed implants, it is noticeable that hybrid latticed implants have lower peak displacements, demonstrating that hybrid arrangements may better limit micro-strains at the bone–implant interface. Smaller micro-strains promote osseointegration by lowering possible micromechanical instabilities that might jeopardize implant life and success. These nuanced micro-deformation behaviors in hybrid implants, characterized by lower peak values and more regulated cyclic changes, highlight their potential for improved clinical performance, especially under dynamic loading circumstances similar to those of normal masticatory activities.

## 4. Discussion

The results of our dynamic explicit finite element analyses provide compelling evidence supporting the hypothesis outlined in the introduction. This study presents an innovative dental implant design that incorporates gyroid TPMS lattice components precisely placed by use of additive manufacturing. The gyroid lattice is positioned in the very middle region of the hybrid lattice design, whereas the fully latticed design extends it to both the neck and middle sections. To assure initial stability, both designs have a square-threaded section at the base. Our dynamic explicit finite element analysis demonstrates that these improvements enhance stress distribution as osseointegration interfaces. The square-threaded region increases implant stiffness, and the gyroid lattice decreases stress concentrations in key locations, like the implant neck and surrounding bone. Furthermore, it improves micromotions under cyclic masticatory stress. These improvements are critical for long-term implant success because they ensure mechanical stability and optimal load distribution. Fully solid implants are frequently associated with increased marginal bone loss and less bone-to-implant contact, which reduces their long-term stability. Hybrid dental implants with lattice structures and screw threads optimize stress distribution, implant stability, and bone integration [4,56,74,77].

Figure 6 shows the modeling of an oblique dynamic load of 118.2 N applied throughout three cycles of masticatory loading at a frequency of 2 Hz for 1.5 s. This technique is consistent with those used in earlier studies using comparable loading settings, verifying the accuracy of the results. Multi-point constraints (MPC) properly mimic mesiodistal, buccal–lingual, and apical load orientations, resulting in a realistic replication of masticatory scenarios. This innovative simulation accurately captures stress and strain distributions under dynamic loading, giving reputable data on implant performance. The findings emphasize the importance of dynamic loading models in connecting theoretical predictions to clinical reality, providing a reliable tool for the preclinical evaluation and optimization of dental implant designs. This demonstrates the simulation tools’ ability to reliably predict clinical performance and validates their effectiveness. This alignment emphasizes the importance of dynamic loading models in the preclinical evaluation of dental implants, providing the groundwork for future breakthroughs in implant design and testing [49,57].

The mesh sensitivity study has revealed that element size has little to no impact on final outcomes between 0.1 mm and 0.15 mm. The highest stress values were essentially equal, demonstrating that finer mesh sizes greater than 0.1 mm do not considerably enhance accuracy. As a result, a mesh size of 0.1 mm is adequate for accurate stress predictions, with a peak stress of 396 MPa. This option achieves the perfect interaction between computational economy and accuracy, making it excellent for the finite element analysis of latticed dental implants with a 333-cell size.

Figure 8 and Figure 9 demonstrate the significance of medium cell sizes in maintaining biomechanical stability in dental implants. The FLI_222 fully latticed implant has an ideal stress distribution (422.34 MPa), but the HI_222 hybrid balances stress at 504.43 MPa. Non-linear designs, with HI_111 exhibiting greater stress than HI_222 and HI_333, emphasize the intricate link between lattice structure and stress. Medium cell sizes optimize micromotions, and increase osseointegration and stress distribution, lowering potential risks associated with early healing. These designs increase the lifespan of the implant while decreasing the danger of bone resorption and mechanical failure. The findings are consistent with those of past studies, highlighting the significance of improved lattice geometries in enhancing stability and outcomes, especially in situations of low bone quality or severe loading. Previous studies found that optimized lattice architectures increase stress distribution and implant lifetime [70,71,72,73,74,75]. These new results support the idea that medium lattice topologies may improve mechanical stability. Furthermore, another study reported that hybrid designs in dental implants had an advantage in terms of biomechanical performance due to cell size optimization [71,73]. The current research expands on this information by revealing that HI_222 is a clinically ideal option, notable for minimizing implant failure and improving patient outcomes.

Figure 10 and Figure 11 show a striking comparison of maximum von Mises stresses in fully and hybrid gyroid latticed dental implants and their accompanying retention screws at various cell sizes. In fully latticed implants, stress reduces consistently with increasing cell size; the smallest implant, FLI_111, showed the maximum stress, at 460.25, MPa, which reduces to 410.86 MPa for FLI_222 and 395.67 MPa for FLI_333 (refer to Figure 10a). This pattern indicates that medium cell sizes improve stress distribution, possibly increasing the implant’s mechanical stability and function. In the case of the hybrid gyroid TPMS lattice design, there is a clear trend of decreasing maximum von Mises stress as the cell size increases. Specifically, the HI_111 model has a maximum stress of 410.25 MPa, which reduces to 362.98 MPa for HI_222 and 341.60 MPa for HI_333 (refer to Figure 10d–f). This pattern shows a continuous decrease in stress with increasing cell size. Similarly, the accompanying retention screws follow a similar pattern, with stress decreasing from 452.68 MPa in FLI_111 to 387.22 MPa in FLI_333, demonstrating how cell size optimization may improve overall implant system integrity (refer to Figure 11a–c). This arrangement not only enhances the implant’s functional lifetime by preventing mechanical failures, but it also protects accompanying components such as retaining screws and surrounding bones. Hybrid latticed implants find the best balance between porosity and strength by increasing cell size and adding solid structural elements. This leads to new implant designs that meet both biomechanical and clinical standards. In contrast to prior studies that suggested lower stress at the implant neck for porous designs [78], our data show that tension is highly concentrated in the lattice and neck hole areas in both completely and hybrid latticed implants. This approach emphasizes the need for improved design considerations in these crucial areas.

Figure 12 and Figure 13 show stress distribution at the bone–implant contact, which has significant clinical relevance. Among fully latticed designs, the FLI_333 model showed the highest stress concentration in cortical bone, increasing the possibility of mechanical failure in load-bearing locations. In contrast, the FLI_222 design increased stress distribution, lowering biomechanical challenges like bone resorption and implant instability. Hybrid designs improved performance even further, with HI_222 exhibiting the greatest stiffness-to-flexibility ratio. By combining lattice structures with solid components, this arrangement significantly reduced stress and enhances implant lifespan. Clinically, these findings highlight the effectiveness of hybrid implants like HI_222 in minimizing localized bone loss and increasing outcomes under difficult settings such as poor bone quality or heavy loading. These findings are consistent with those of previous research highlighting the importance of optimal lattice and hybrid designs in ensuring long-term biomechanical stability and implant success. This characteristic effectively reduces peak stress concentrations commonly found around the holes of cortical and cancellous bones, aligning with prior research that pinpoints potential sites for bone resorption [66,71,78].

Figure 14 and Figure 15 depict stress distribution in the crowns and abutments of fully and hybrid lattice implants, which has substantial medical relevance. Fully latticed implants, such as FLI_111, have significant abutment stresses (504.43 MPa), which might cause mechanical fatigue under clinical loads. In contrast, hybrid designs, notably HI_222, lower abutment stress to 251.17 MPa, increasing implant longevity. Clinically, this stress decrease reduces the chance of abutment failure and boosts implant life. Previous research has shown that integrating lattice systems with solid parts improves biomechanical performance and reduces problems such as abutment loosening or fracture.

Figure 16 and Figure 17 show the micro-strain responses of completely and hybrid gyroid TPMS lattice implants to cyclical masticatory stress for 1.5 s. Fully latticed implants, especially FLI_333 and FLI_222, showed larger micro-strains (1100 µm and 1095 µm, respectively), suggesting more flexibility but increased micromotion, which could inhibit osseointegration. Hybrid designs lowered the micro-strains substantially, with HI_333, HI_222, and HI_111 peaking at 225 µƐ, 190 µƐ, and 160 µƐ, respectively, exhibiting greater biomechanical stability. With hybrid designs providing greater displacement control and enhanced potential for osseointegration during dynamic mastication, our findings emphasize the impact of lattice structure and cell size on implant function, making them more appropriate for long-term clinical success.

We have compared our results to those of prior research on lattice-based implants made using additive manufacturing (AM). Barba et al. [79] emphasized the need to match lattice mechanical characteristics to bone in order to improve osseointegration and minimize stress shielding. Burton et al. [80] confirmed that optimized lattice geometries make structures more mechanically stable. This fits with our findings that gyroid lattice implants make stress distribution better and reduce micromotion. Similarly, Wang et al. [81] found that 60–70% porous lattices increase bone ingrowth, which supports our design’s pore connection for better integration. These results support the biomechanical viability of gyroid lattice-based dental implants.

The additive fabrication of lattice-structured orthopedic implants reduces weight and improves mechanical performance. However, the longevity of these implants under biological conditions such as corrosion and fatigue, as well as the intricacy of their manufacture, present hurdles. Kladovasilakis et al. [82] stated that addressing these challenges will be critical to their effective clinical utilization.

While additively made implants, especially those with unique designs, have shown encouraging clinical results, questions remain about their long-term biological function. Studies by Anitua et al. [83] reveal good implant survival rates in the short term, but emphasize the danger of problems such as soft tissue exposure and infection, which might possibly be connected to the implant’s complicated design. According to Roy et al. [82], the greater complexity of these designs may result in higher production costs, which may impact the broad acceptance of these implants in clinical settings. Nonetheless, as progress is made and more data are acquired, these issues may be addressed via new materials and production methods. As a result, more long-term studies are required to establish the overall practicality of these implants in clinical settings.

The current work applies dynamic explicit finite element analysis to completely and hybrid-latticed dental implants with a square-threaded design in the implant’s bottom area. While the findings are impressive and provide important insights into clinical and customized dental implant design, further studies are required before use in clinical trials. Future research should focus on creating the implants utilizing metal additive manufacturing techniques, such as powder bed fusion with titanium, to assess their fatigue resistance. In vivo testing is also advised to confirm their performance under physiological settings. In summary, the results underscore the critical role of lattice cell size and hybrid design in optimizing mechanical stability and stress distribution. Hybrid latticed implants, particularly the HI_222 model, outperform other designs by reducing mechanical stresses, minimizing micromotions, and enhancing osseointegration. These findings offer valuable insights for advancing implant designs to meet both biomechanical and clinical requirements, paving the way for improved patient outcomes and long-term implant success.

## 5. Conclusions

This research investigates the biomechanical performance of dental implants, with an emphasis on the effects of lattice structure and cell size on stress distribution, mechanical stiffness, and micromotion stability. The study’s goal is to improve clinical outcomes by comparing fully gyroid latticed and hybrid gyroid latticed implants with a solid neck across various cell sizes. The investigation came to the following key conclusions:

▪ The square-threaded region improves initial implant anchoring, facilitating quicker osseointegration and longer-term retention under functional stresses;
▪ Hybrid latticed implants, especially those with medium cell sizes (HI_222), exhibit excellent stress distribution. These designs efficiently lower stress concentrations in crucial locations, possibly improving implant lifetime and success;
▪ The additional thread effectively distributes load stress and reduces micromovements at the implant–bone contact, maintaining stability and decreasing pressure on the surrounding bone for long-term integration;
▪ In comparison to fully latticed implants, hybrid designs provide higher mechanical rigidity. This improvement is critical for preserving structural integrity under typical masticatory stresses, which is necessary for the implants’ long-term durability;
▪ The researchers discovered that hybrid latticed implants with solid necks had significantly reduced micromotions at the bone–implant contact. This decrease is critical for improving osseointegration and lowering the possibility of implant failure due to biomechanical instabilities;
▪ Tailored hybrid latticed designs with optimal cell sizes show promise in clinical applications, outperforming standard fully latticed implants. The HI_222 arrangement is especially advantageous, offering a mix of flexibility and load-bearing capability appropriate for a wide range of clinical conditions;
▪ Adding a solid neck to hybrid latticed implants makes stress distribution much better, managing stress in a way that a fully latticed configuration cannot. Medium-sized cells, particularly in hybrid topologies like HI_222, function optimally, establishing new standards in dental implant design by combining light weight with strong mechanical characteristics;
▪ This research demonstrates the efficacy of additive manufacturing for producing biomechanically optimized dental implants. These improved manufacturing procedures substantially enhance patient outcomes by precisely matching bone’s inherent biomechanical characteristics, indicating an optimistic future for dental implant technology.

These findings emphasize the need for rigorous design and material selection in the creation of dental implants in order to fulfill the stringent demands of clinical application. The study’s results provide a basis for future research and development in dental implant technologies, with the goal of achieving optimum biomechanical compatibility and performance.

## Data Availability

The original contributions presented in the study are included in the article, and further inquiries can be directed to the corresponding author.
